# Differences in Merkel cell carcinoma between Hispanics and Whites: A 21-year SEER database study

**DOI:** 10.1016/j.jdin.2025.05.012

**Published:** 2025-06-19

**Authors:** Jenna Koblinski, Mitchell A. Taylor, Jennifer M. Fernandez, Collin M. Costello

**Affiliations:** a Department of Dermatology, Emory University School of Medicine, Atlanta, Georgia; b Creighton University School of Medicine, Omaha, Nebraska; c Department of Dermatology, University of Nebraska Medical Center, Omaha, Nebraska; d Department of Dermatology, Mayo Clinic, Scottsdale, Arizona

**Keywords:** cutaneous oncology, ethnicity, health care differences, Merkel cell carcinoma, mortality, SEER database, skin cancer, survival

*To the Editor:* Merkel cell carcinoma (MCC) is a rare, aggressive dermatologic malignancy. This tumor has been associated with the Merkel cell polyomavirus, ultraviolet exposure, immunosuppression, and older age.^1^^,^^2^ While racial differences have been noted for MCC,^3^ there is a paucity of data on ethnic differences in MCC between White Hispanic (WH) patients and non-Hispanic White patients (NHW), especially when looking at disease-specific survival.^4^ A recent study of the National Cancer Database found that Hispanic patients with MCC have lower mortality compared to non-Hispanic patients; however, the National Cancer Database does not collect data on cause-specific death.^4^ Our study aimed to evaluate MCC differences between WH and NHW patients.

The Surveillance, Epidemiology, and End Results database was queried to identify WH and NHW patients diagnosed with biopsy-confirmed cases of cutaneous MCC (International Classification of Disease for Oncology third edition histology code 8247/3; primary site code C44.0-44.9) from 2000 to 2021. Statistical analyses were completed using SPSS version 29.0.2, including Chi-square and Fischer’s exact tests, Mann–Whitney U test, Kaplan–Meier and log-rank tests, and multivariable Cox regressions (statistical significance *P* < .05).

A total of 10,722 patients were identified; the majority were NHW (94.0%), male (64.1%), aged 80+ (41.4%), diagnosed with localized disease (62.3%), and presented with primary tumors of the head and neck region (47.3%). Clinicopathologic features can be found in Table I. There was a significant association between ethnicity and disease stage (*P* < .001), where WH patients were diagnosed with higher rates of distant-stage disease (13.2% vs 9.7%) compared to NHW patients. There was also a significant association between ethnicity and primary tumor location (*P* < .001), where NHW patients exhibited higher rates of head and neck primary tumors (47.9% vs 37.1%), while WH patients presented with higher rates of upper extremity (31.3% vs 26.7%), truncal (10.4% vs 10.2%), and lower extremity tumors (21.1% vs 15.1%). WH patients exhibited a larger median tumor size of 18.0 mm (interquartile range (IQR) 10.0-30.0) versus 15.0 mm (IQR 8.0-26.0) in NHW patients (*P* = .035). Univariable Kaplan–Meier analysis revealed significantly reduced 5- and 10-year DSS rates in NHW patients (52.0% and 49.0%, respectively) compared to their WH counterparts (66.0% and 62.0%) (*P* < .001) (Fig 1). Multivariable analysis adjusting for age, sex, income, rural-urban living, primary tumor location, disease stage, and tumor size revealed NHW ethnicity was independently associated with a +37% increased disease-specific mortality risk (adjusted hazard ratio: 1.37; 95% CI 1.06-1.78) compared to their WH counterparts.

**Table I:** Clinicopathologic features of Hispanic and Non-Hispanic White patients diagnosed with Merkel cell carcinoma

*n* = 10722	Non-Hispanic White (*n* = 10077)	Hispanic White (*n* = 645)	*P*-value
Age at diagnosis (y)			**<.001**∗
<40	22 (0.2%)	5 (0.8%)	
40-49	164 (1.6%)	21 (3.3%)	
50-59	718 (7.1%)	87 (13.5%)	
60-69	1796 (17.8%)	149 (23.1%)	
70-79	3106 (30.8%)	213 (33.0%)	
80+	4271 (42.4%)	170 (26.4%)	
Sex			**<.001**†
Male	6561 (65.1%)	310 (48.1%)	
Female	3516 (34.9%)	335 (51.9%)	
Annual income‡			.188†
<$75,000	4212 (41.8%)	287 (44.5%)	
$75,000+	5865 (58.2%)	358 (55.5%)	
Rural-urban living§			**<.001**†
Urban	8709 (86.4%)	618 (95.8%)	
Rural	1368 (13.6%)	27 (4.2%)	
Marital status			**.003**†
Married	5775 (63.4%)	324 (57.1%)	
Not married	3328 (36.6%)	243 (42.9%)	
Primary tumor location			**<.001**∗
Head and neck	4450 (47.9%)	218 (37.1%)	
Upper extremity	2479 (26.7%)	184 (31.3%)	
Trunk	950 (10.2%)	61 (10.4%)	
Lower extremity	1402 (15.1%)	124 (21.1%)	
Disease stage at presentation‖			**.027**∗
Localized	5053 (62.5%)	324 (59.4%)	
Regional	2247 (27.8%)	149 (27.3%)	
Distant	782 (9.7%)	72 (13.2%)	
Median tumor size, mm (IQR)	15.00 (8.00-26.00)	18.00 (10.00-30.00)	**.035**¶
Surgery#	8271 (82.4%)	511 (79.7%)	.086†
Chemotherapy#	1036 (10.3%)	89 (13.8%)	**.006**†
Radiation#	4676 (47.2%)	311 (48.7%)	.437†

Significant *P* values (<.05) are in bold.

*IQR*, Interquartile range; *SEER*, Surveillance, Epidemiology, and End Results.

∗ Test statistic calculated using Chi square test.

† Test statistic calculated using two-sided Fisher’s exact test.

‡ Annual income data reported from the census tract incomes; not reported as individual or family incomes.

§ Rural vs urban living was determined using the SEER Rural-Urban Continuum codes initially defined by the Economic Research Service at the United States Department of Agriculture.

‖ The SEER historical staging system categorizes malignancies into 3 groups: localized (restricted to the organ of origin), regional (spreading beyond the originating organ or involving nearby lymph nodes), and distant (having spread to a distant part of the body through direct extension, non-contiguous metastasis, or via the lymphatic system to distant lymph nodes). This classification framework is applicable across a broad spectrum of malignancies and accommodates variations in formal staging systems over time.

¶ Test statistic calculated using Mann–Whitney U test.

# Compared to not receiving this treatment. Chemotherapy includes immunotherapy.

**Fig 1:**
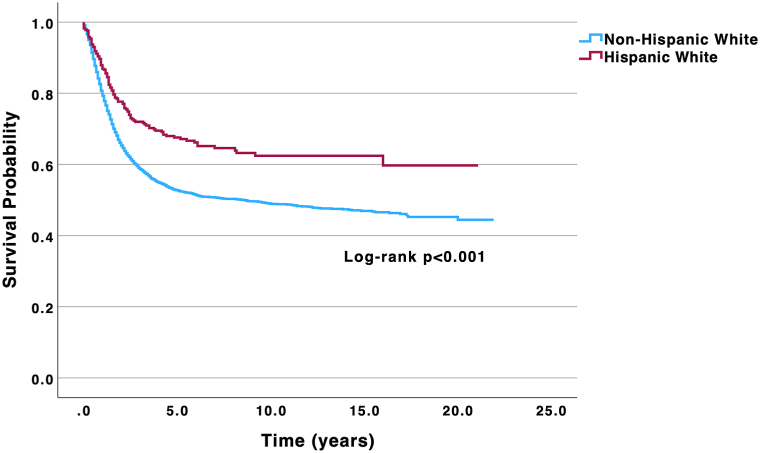
Univariable Kaplan–Meier analysis highlighting enhanced 5- and 10-y disease-specific survival rates in Hispanic White patients compared to non-Hispanic White patients.

While WH patients have significantly higher rates of distant disease at presentation, NHW patients had worse disease-specific survival with MCC. This suggests biological differences in MCC between WH and NHW. Prior studies have shown that UV-induced MCC tumors have worse outcomes than viral-induced tumors, so perhaps the improved outcomes in WH are due to a higher percentage of viral-induced tumors, a variable not collected in the Surveillance, Epidemiology, and End Results database.^5^ While head and neck MCC, tumor diameter, and distant stage are associated with poorer outcomes, these were controlled for in the multivariable analysis. Further evaluation of differences in tumor biology between WH and NHW could help us better understand MCC disease.

## Conflicts of interest

None disclosed.
